# Interventions to improve health literacy among Aboriginal and Torres Strait Islander Peoples: a systematic review

**DOI:** 10.1186/s12889-021-10278-x

**Published:** 2021-01-30

**Authors:** Simone Nash, Amit Arora

**Affiliations:** 1 School of Health Sciences, Western Sydney University, Locked Bag 1797, Penrith, NSW 2751 Australia; 2 Translational Health Research Institute, Western Sydney University, Locked Bag 1797, Penrith, NSW 2751 Australia; 3 Discipline of Child and Adolescent Health, Sydney Medical School, Faculty of Medicine and Health, Westmead, NSW 2145 Australia; 4 Oral Health Services, Sydney Local Health District and Sydney Dental Hospital, Surry Hills, NSW 2010 Australia

**Keywords:** Aboriginal, Torres Strait Island, Indigenous, First Nations Peoples, Health literacy, Australia, Social determinants of health, Socio-economic inequality, Systematic review

## Abstract

**Background:**

Aboriginal and Torres Strait Islander peoples continue to experience poorer health outcomes than other population groups. While data specific to Indigenous Australians are scarce, a known social health literacy gradient exists linking low health literacy and poor health outcomes within many minority populations. Improving health literacy among Indigenous Australians is an important way to support self-determination and autonomy in both individuals and communities, by enhancing knowledge and improving health outcomes. This review aims to rigorously examine the effectiveness of health literacy interventions targeting Aboriginal and Torres Strait Islander peoples.

**Methods:**

A systematic review across six databases (The Cochrane Library, PubMed, Embase, SCOPUS, ProQuest Dissertation and Thesis and Web of Science) was performed for publications evaluating interventions to improve health literacy among Indigenous Australian adults using search terms identifying a range of related outcomes.

**Results:**

Of 824 articles retrieved, a total of five studies met the eligibility criteria and were included in this review. The included studies evaluated the implementation of workshops, structured exercise classes and the provision of discounted fruit and vegetables to improve nutrition, modify risk factors for chronic diseases, and improve oral health literacy. All interventions reported statistically significant improvement in at least one measured outcome. However, there was limited involvement of the Aboriginal and Torres Strait Islander community members in the research process and participant retention rates were sub-optimal.

**Conclusion:**

There is limited evidence on interventions to improve health literacy in Indigenous Australian adults. Participation in interventions was often suboptimal and loss to follow-up was high. Future studies co-designed with Aboriginal and Torres Strait Islander community members are needed to improve health literacy in this population.

**Supplementary Information:**

The online version contains supplementary material available at 10.1186/s12889-021-10278-x.

## Background

Health literacy is a social construct which can be defined as the skill required to perform tasks such as reading, writing, understanding, and interpreting the basic health information and services in order to make appropriate health decisions [1]. The term encompasses not only a range of cognitive skills which allow people to access and utilise health information to enhance their ability to engage in promotion and maintenance of good health [1, 2]; but also the skills required for the incorporation of of health information, navigation, processing, problem-solving and decision-making [3]. Health literacy, a term first proposed in the 1970’s, is a concept that continues to evolve and be redefined. The Calgary Charter on Health Literacy was formulated by the World Health Organization and defines the term as the ability of both the public and healthcare professionals to locate, comprehend, share and utilise health information [4]. A recent systematic review investigating the meaning of health literacy defined it as the ability of an individual to obtain and translate knowledge and information in order to maintain and improve health in a way that is appropriate to the individual and system contexts [5]. Health literacy encompasses all the skills which contribute to the ability to live a healthful life [3]. In this respect, health literacy is recognised as an important factor that not only involves the patient/consumers’ and the individuals/organisations involved in the provision of care [6]; but is also a concept that pertains to broader societal health practice and the ways in which health information is sought and shared among population groups (distributed health literacy) [7, 8].

Health literacy is a determinant of health [9], and is conceptualised on three hierarchal levels: functional, communicative/interactive and critical; beginning with basic skills through to the most complex which include the ability to critically analyse health information and affect change on the health of self and others [10]. Health literacy is critical to health promotion and encompasses the environmental, political and social factors which either impede or improve health outcomes [11]. A social gradient related to health literacy has been identified in all national health surveys [9] linking low levels of health literacy levels with poorer health outcomes among minority population groups [12].

The understanding of the concepts of health literacy gains deeper meaning in the context of culture. Cultural health beliefs affect how people think and feel about their health, when and from whom they seek health care, and how they respond to prevention and management of health conditions. Definitions of health literacy that do not recognise the potential effects of culture on the communication and understanding of health information neglect deeper interpretations of what it means to be health literate [10]. This is especially important given the ethnic and linguistic diversity in Australia with the urgent need to improve cultural literacy of service providers [13].

Aboriginal and Torres Strait Islander peoples are the First Peoples of Australia and comprise about 3.3% of the Australian population [14, 15]. Many groups of Aboriginal and Torres Strait Islander peoples do not receive the same opportunity to achieve and maintain physical and mental health as non-Indigenous Australians [16]. The overall burden of disease in Indigenous populations remains more than twice that of their non-Indigenous counterparts, with prevalence of chronic diseases including mental illnesses, respiratory, renal and cardiovascular diseases of particular concern [17]. Life expectancy for many Aboriginal and Torres Strait Islander Australians remains around 17 years lower than that of other Australians [18], with a recent report showing no improvement in these statistics in the last decade [19]. The economic cost of the burden of disease is substantial, and while almost 50% more per capita is spent on Indigenous health, [12, 20] spending falls well short for the significantly more complex needs of this population [20]. Though progress has been made in recent years to improve the health and well-being of Aboriginal and Torres Strait Islander peoples [19], advancements in health outcomes within the general population has meant that the gap between the health of Indigenous and non-Indigenous Australians continues to widen [19, 21].

The reasons for continued Indigenous health disparities are numerous and complex and begin with the profound and enduring impact of colonisation and subsequent dilution of language, culture, disconnection from country, systemic discrimination and mistreatment [22]. The holistic understanding of health and wellbeing for Indigenous Australians involves the whole community throughout the entire life-course and includes broad issues such as social justice, equity, and rights, as well as traditional knowledge, traditional healing, and connection to Country [23]. A number of theories have been proposed to demonstrate relationships between health literacy, health outcomes and Indigeneity [24]. To date, these theories lack the support of data, though understanding the potential relationships that may be at play is an important strategy in determining where intervention might be effective [24]. The key components of the health literacy relationship pathways outlined by Australian Commission on Safety and Quality in Health Care [24] include: how individuals’ access and utilise health services; the interactions that occur between consumers and providers; and how people manage and exert control over their own health [24]. These health literacy pathways are reflected in the outcome measures used in this review (knowledge or skills; attitudes, motivation or behaviour changes; self-efficacy; self-management; engagement with and use of available health care services; health status/outcomes).

Australian State and Territory Health Services continue to attempt to address the issues impacting these pathways and the social determinants of Indigenous health, including the continuing need for accessible and culturally appropriate health services for Aboriginal and Torres Strait Islander peoples. In 2007, The Council of Australian Governments (COAG) identified Closing the Gap as a national priority, and though progress has been made in some domains, many require more work and unfortunately the status of others have worsened [25]. The recently published Closing the Gap Report 2020 highlights the struggle to meet these targets continues to this day [26]. The reasons for the shortcomings of this initiative are many, and include unrealistic targets, inadequate Indigenous involvement and other issues of mismanagement [18]. It is worth noting that while the social determinants of Indigenous health remain unchanged, reaching such targets will remain unlikely [18].

Understanding health literacy and its relationship to the status of Indigenous health is an important step in working toward promoting health literacy in Aboriginal and Torres Strait Islander peoples [27]. The 2006 Australian Bureau of Statistics (ABS) survey [28] and the 2018 Australian National Health Survey [29] did not report on health literacy data related of Aboriginal and Torres Strait Islander peoples. However, it is thought to be very likely that health literacy levels among Aboriginal and Torres Strait Islander peoples are lower than among other non-Indigenous population groups [24]. In recent years, several systematic reviews have been conducted both in Australia and internationally examining interventions for individuals with low health literacy [16, 30, 31], with most of these targeting specific sub-groups. Other systematic reviews have examined the interventions to improve health literacy among Indigenous people affected by cancer [32] or evaluated health literacy interventions among minority populations more broadly [31, 33–35]. To date, research investigating Aboriginal and Torres Strait Islander peoples and limited health literacy levels is sparse, and more is required in order to increase awareness of the issue in order to increase information, access and resources. To the best of our knowledge this is the first systematic review to evaluate the effectiveness of health literacy interventions targeting Indigenous Australians and aims to add knowledge through the synthesis of available evidence related to interventions to improve health literacy among Indigenous Australian adults.

## Methods

This review was conducted based on the Preferred Reporting Items for Systematic Reviews and Meta-analysis (PRISMA) guidelines [36]. The protocol for this review is published and registered with The PROSPERO International Register of Systematic Reviews (PROSPERO 2020: CRD42020130529) [37].

### Eligibility criteria

The inclusion and exclusion criteria were formulated based on the Population, Intervention, Comparator, Outcome and Study Design (PICOS) framework [38] (Table 1).

**Table 1:** Eligibility for inclusion in the systematic review

Inclusion Category	Inclusion Criteria
Population	- Identify as Aboriginal and/or Torres Strait Islander people - > 18 years old
Intervention	Includes improvement of a health outcome of interest: - Health literacy - Knowledge or skills - Attitudes/Motivation - Behaviour change - Self-efficacy - Self-management - Engagement with/Use of services - Health status
Study Design	Experimental designs - Randomised Control Trials - Quasi-randomised Control Trials - Matched Comparison Groups - Controlled Before and After - Pre/Post - Interrupted Time Series
Publication	English only Reports quantitative measurements

#### Types of participants

To be considered for inclusion, studies must have been conducted with adult Indigenous Australian participants over 18 years of age. In studies recruiting Indigenous Australians and participants of other backgrounds, data specific to Indigenous Australians was sought from the author/s. Where no such data were obtainable such studies were excluded. No restriction was made regarding the gender of participants.

#### Types of interventions

Any intervention that the authors reported to be aimed at improving health literacy were included. This may include interventions that aimed to change any predefined aspect of health literacy in an individual or population, including:

- Knowledge about risk factors, disease, prevention and/or treatments
- Attitudes, confidence or beliefs
- Ability to care for self (including disease self-management), self-efficacy and autonomy
- Health-related literacy skills
- Help-seeking behaviours, awareness, access to or utilisation of health care services
- Engagement with and cultural safety of health care services and health professionals

#### Types of comparators

Comparator/control groups included non-intervention or usual care group, alternative interventions, matched samples receiving the same intervention and historical control groups.

#### Types of outcome measures

Studies that described an outcome measure related to health literacy were included in this review. These outcome measures may have included:

- Validated health literacy measurement tool
- Knowledge or skills
- Attitudes, motivation or behaviour changes
- Self-efficacy
- Self-management
- Engagement with and use of available health care services
- Health status/outcomes

#### Types of studies

All studies describing an intervention with one of the following designs were included in this review: Randomised control trials (RCTs), quasi randomised control trials, matched comparison group designs, controlled before and after studies, pre-post-test studies or interrupted time series. Qualitative studies and conference proceedings were excluded from this review. In cases where more than one paper was published by the authors detailing the same study, relevant data from all papers were extracted as one study.

### Information sources

The search began in consultation with a professional Health Sciences librarian, who assisted in guiding the authors to relevant databases. The following databases were subsequently searched: Cochrane Central Register of Controlled Trials (CENTRAL) (The Cochrane Library), MEDLINE (OVID) and Embase (OVID). Additionally, Proquest Dissertation and Thesis, SCOPUS and Web of Science were searched for eligible grey literature. Furthermore, a parallel manual search of the reference lists of all eligible studies and previously published systematic reviews on health literacy was performed. No restriction was made on the date of publication. The initial search was concluded on the 24th April 2019, was re-performed on the 14th October 2019 and subsequently updated on the 20th June 2020. Studies were restricted to English language only.

### Search strategy

The Population Intervention Comparator Outcome and Study Design (PICOS) framework [38] was used to devise the search terms. A combination of Medical Subject Headings (MeSH) terms and keywords were formulated in consultation with a professional Health Sciences Librarian and peer-reviewed to ensure completeness. These terms included all identified variations of “Australian Aboriginal peoples,” “Indigenous Australians” and “Torres Strait Islander people” combined with all identified headings and suggested terms for “health literacy,” “health education,” “access to information,” “consumer health information,” “patient education,” “health knowledge, attitudes and practice,” “self-care,” “self-concept” and “self-efficacy.”

Combinations of keywords and terms using Boolean operators, truncation, phrase searching, and Medical Subject Heading (MeSH) were used in the search strategies. The initial search string was developed and tested using Embase (OVID) (see search strategy Additional file 2). This search was subsequently adapted to the syntax and subject headings of the other databases employed.

### Study selection

Studies identified through database searches, grey literature, theses and manual searches were subsequently exported to EndNote X9 [39] for removal of duplicates, screening and selection. Two reviewers (SN and AA) independently screened the articles based on the eligibility criteria. Abstracts of the studies that were considered to potentially meet the criteria for this review were read by two reviewers (SN and AA). Full texts of studies considered to be eligible were then read by two reviewers (SN and AA). Study authors were contacted to seek additional information in case of any uncertainty on eligibility. A total of two attempts were made to contact the study authors, and if no response was received studies were screened for eligibility based on the information available. Tabulated details of the studies that were read in full and subsequently excluded have their reason/s for exclusion reported in Additional file 3. The study selection process was carried out with reference to the Preferred Reporting Items for Systematic Reviews and Meta-Analysis (PRISMA) checklist [36] (Additional file 1).

### Data collection process

A standardised data extraction form was developed and pilot-tested based on a checklist presented in The Cochrane Handbook for Systematic Reviews of Interventions [40]. Data were extracted for one study initially to ensure consistency across reviewers and ensure all relevant data were captured. Data from all the included studies were extracted independently by two reviewers (SN and AA). The data extracted from each included study included information on the first author, year of publication, information on the participants/sample, follow-up period, particulars of the intervention/s, study setting, design, funding, data analysis techniques, as well as a brief summary of outcome measures, results and conclusion for each study. For studies where necessary data were missing, the corresponding author/s were contacted with a maximum of two attempts to seek clarification. Where no response was received, data extraction was completed using the information available. The completed data summary table is included here (Table 2).

**Table 2:** Summary of included studies

Source, Design, and Setting	Aim/s	Participants and Follow-up period	Intervention	Outcome Measures	Results	Conclusions	Funding
Brimblecombe, et al. [41, 42] Stepped-wedge RCT followed by Longitudinal sub-study Extremely remote communities of Northern Territory	Examine the impact of a 20% price discount on fruit, vegetables, water, and artificially sweetened soft drink purchases +/− consumer education Assess impact of the intervention on mediators, moderators and consequent dietary behaviour	20 communities where the community store was managed by the ALPA or OBS with no alternative food outlet present within a 20 km radius Community populations of at least 100 persons (most approx. 200–400 persons) with estimated total population 8515 persons Also assessed randomly selected adult ‘primary soppers’ from 5 communities via 3x questionnaire (on iPad) over 48-week period -T1 baseline (*n* = 148) -T2 immediately post intervention (*n* = 85) -T3 24-weeks post intervention (*n* = 73) − 92% female respondents -Questionnaire completion incentivised with $20 gift of fruit, vegetables and water	All stores: 20% discount on all fresh and frozen fruit & vegetables (excluding potato products), bottled water and artificially sweetened soft drinks promoted in store and applied at point of sale Discounts applied over 24-week period 0 stores: 10 stores (2 in each set) randomly assigned to receive consumer education (with at least 1 ALPA and 2 OBS in each set) Consumer nutrition education delivered in-store using posters, activities, demonstrations and prizes Delivered over 24-week period Different themes each month Use of Social Cognitive Theory	Weight of fruit and vegetables purchased per capita (in grams) Weight of water and artificially sweetened soft drinks per capita (in grams) Weight of less healthy foods, regular soft drinks and other beverages per capita (in grams) Daily intake of fruit (g), vegetables (g), water (mL), regular soft drink (mL) and diet soft drink (mL) Percieved affordability of fruit and vegetables Self-efficacy to positively change intake, cook and try new foods	12.7% (95% CI, 4.1–22.1) increase in purchase of both fruit and vegetables Largest change in fruit purchases 20.6% (95% CI, 6.8–36.2) as opposed to 9.0% for vegetables (1.2–17.4) Consumer education further increased purchase of fruit and vegetables 7.6% (95%CI, 3.6–20.2) with greatest benefit seen in purchase of vegetables 13.6% (95% CI, 2.6–25.7) Other food purchases also increased, including those of less-healthy foods 13.4% (95%CI, 1.7–26.4) Perceived affordability of fruit and vegetables was associated with positive changes in dietary intake	Discounting fruits and vegetables by 20% to help protect against obesity and diet-related disease is partially effective The effects of fiscal interventions can be enhanced by the use of creative merchandising techniques and consumer education The intervention was partially effective in increasing consumption of fruits and vegetables among the target population, but was not strong enough to overcome mediators and moderators affecting lasting behaviour change	NHMRC
Canuto, et al. [43] Pragmatic RCT Urban South Australia (Adelaide)	Evaluate the effectiveness of intervention on reducing cardio-metabolic risk in population of interest Assess whether outcomes maintained at 3 months post intervention	100 Indigenous women with waist circumference > 80 cm Aged 18–64 years Followed up over approx. 6 months with measurements taken at intervals: - pre-intervention (T1) - immediately post 12-week intervention (T2) - 3 months post intervention (T3) Poor participation at classes and workshops	12-week structured exercise and nutrition education program The program comprised: - bi-weekly 1-h group exercise classes (aerobic and resistance training by female instructor) - incidental activity and walking measurement (pedometer and exercise diary) - nutrition workshops (4 × 1 h by female dietician) - positive reinforcement and encouragement (fortnightly newsletters)	Anthropometric and Biomedical Measurements: -Body weight -BMI -Hip and waist circumference -BP -Fasting glucose and insulin -HbA1C -Lipid profile -CRP	Statistically significant reduction in weight and BMI in ‘active’ group relative to ‘waitlisted’ group at T3: -Weight: 2.5 kg (95%CI, 4.46–0.54) -BMI: 1.03 kg/m2 (95%CI, 1.79–0.27) Other relative differences in measures noted however none were statistically significant	Modest reductions in weight, BMI and BP evident at T2 with further improvement by T3 No change in primary outcome measures (waist circumference and metabolic measures)	NHMRC
Ju, et al. [44] RCT Port Augusta, Regional South Australia	Determine the effect of an oral health literacy intervention on oral health-literacy related outcomes among rural-dwelling Indigenous Australian adults	400 Indigenous adults residing in Port Augusta or surrounds Case group mostly female, low levels of education, receiving welfare and regular consumers of tobacco and alcohol Randomised into: -Group A *Intervention* (*n* = 203) -Group B *Control* (*n* = 197) Incentive-based participation - gift vouchers were used to compensate participants for their time Overall attendance at workshops just 46.8% (95 participants)	Five 1.5-h workshops over 12 months conducted by Indigenous staff Workshops comprised of presentations, hands-on activities, interactive displays, group discussion and role playing Developed with two Indigenous research officers Use of Bandura’s Social Cognitive Theory Incentive-based intervention model Self-report questionnaires at baseline and 12 months	Some improvement of reported related to: -Knowledge -Skills -Attitudes -Self- Efficacy -Motivation -Activation -Behaviour Change Outcome measures used include HeLD-14, OHIP-14, Lachman and Weaver and Finlayson scales	Improvement of oral health literacy (mean change 1.3, 95% CI) – difference statistically significant Creating awareness of the social impacts of poor oral healt Increasing sense of personal control and oral health self-efficacy Statistically significant improvement in participants who reported ‘water with fluoride is good’ (RR 1.2, 95%CI, 1.1–1.3)	The intervention was found to be partially successful in improving oral health literacy and oral health literacy-related outcomes only after multiple implementations The intervention is considered by the authors to not be feasible in practice, due to poor attendance and expense.	NHMRC
Mills et al. [45] Pre-Post Quasi Experimental Urban areas of South East Queensland	Improve health outcomes of individuals with or at risk of cardiovascular disease Improve self-management Assess effectiveness of intervention on clinical outcome measures	Inclusion of 85 participants Referral by general practitioner Inclusion criteria: -Presence of at least one cardiovascular disease, or -Presence of at least one cardiovascular disease risk factor Age 18–74+ years 12-week follow up period	45-min ‘yarning’ (education) sessions followed by 1-h exercise sessions (both with qualified health professionals and Indigenous staff in attendance)	Changes in clinical outcome measures: -Weight -BMI -BP -Waist & hip circumferences -Results of 6-min walk test	Statistically significant results included: -Reduction in the weight of extremely obese participants (1.6 kg, 0.1–3.0 kg, 95% CI) -Improved distance able to be walked in six minutes (0.053 km, 0.01–0.07 km, 95% CI) -Decreased BP in participants who were hypertensive at baseline (11 mmHg, 3.2–18.8 mmHg, 95%CI)	This short-term study may indicate a possibility for increased improvements in clinical outcomes as the result of behaviour change over longer time periods This program could provide a useful model for similar future interventions among Aboriginal and TSI populations	National Heart Foundation Australian Indigenous Scholarship
Pettigrew, et al. [46] Pre-Post Longitudinal Survey Both metropolitan and regional areas throughout Western Australia	Assess the relative effectiveness of an adult nutrition education program for both Indigenous and non-Indigenous participants Evaluate whether the program requires modification to achieve the same knowledge and behaviour changes in the Indigenous group as the non-Indigenous group	Total sample of 875 Western Australians: − 706 non-Indigenous − 169 Indigenous Pre-post questionnaires followed up over 2- year evaluation period	Two course types were offered to participants: -Single-session course of 1–2 h covering a limited number of nutrition topics -Multi-session courses ranging from 2 to 8 sessions providing information on a broader range of nutrition topics Participants free to choose which best suited their needs	Confidence in ability to buy healthy foods on a budget Knowledge about risk factors and disease Informed food choices and better nutrition Motivation and behaviour change	Improvements in outcome measures greater among Indigenous participants in all instances Improvement in confidence of ability to buy healthy foods on a budget between participants: -Indigenous: (M = 0.74, SD = 1.17, *n* = 156) -Non-Indigenous: (M = 0.53, SD = 1.05, *n* = 676)	The program demonstrated superior outcomes amongst Indigenous participants when compared to non-Indigenous participants on many key outcomes Potential to achieve positive results with broader population while also addressing health inequities for Indigenous population	Western Australian Department of Health

*ALPA* Arnhem Land Progress Aboriginal Corporation, *BMI* Body Mass Index, *BP* Blood Pressure, *CI* Confidence Interval, *CRP* C-Reactive Protein, *HbA1C* Glycated Haemoglobin, *HeLD* Health Literacy in Dentistry Scale, *M* Mean, *NHMRC* National Health and Medical Research Council, *OBS* Out Back Stores, *OHIP-14* Oral Health Impact Profile, *SD* Standard Deviation, *TSI* Torres Strait Islander people

### Assessment of methodological quality

The methodological quality of each study was assessed independently by two reviewers (SN and AA) using the appropriate standardised critical appraisal tool produced by the Joanna Briggs Institute (JBI) [47].

### Data synthesis

Following the tabulation of extracted data, a narrative was created to provide descriptive synthesis of the included studies. Outcomes were described from the data using differences in means, proportions, risk ratios with relevant 95% confidence intervals.

## Results

### Results of the search

**Fig. 1:**
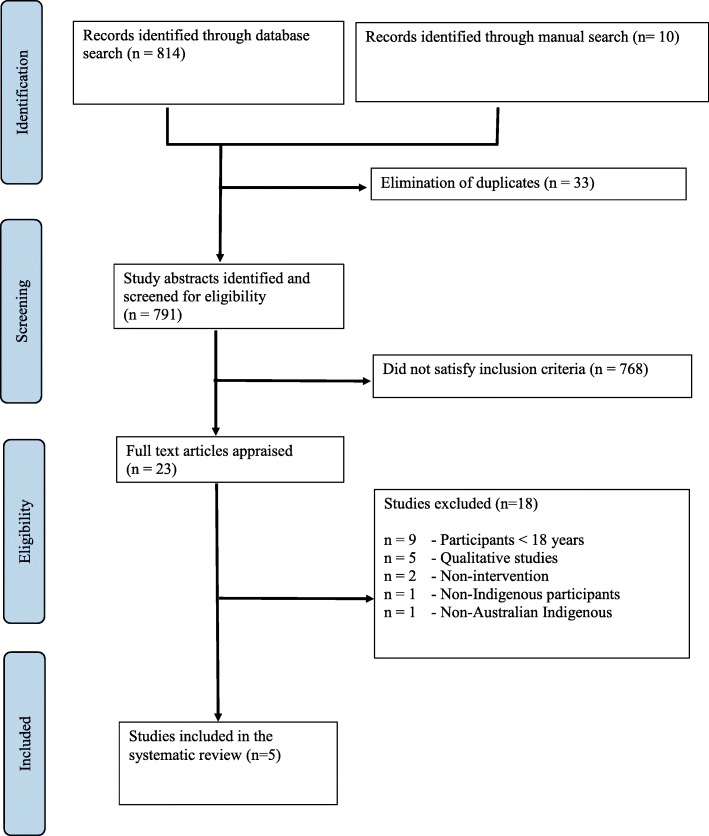
PRISMA Flow Diagram

Initial searches retrieved 824 citations from all databases and manual searches. A total of 791 studies remained following removal of 33 duplicates. A total of 768 studies were excluded as they did not meet the inclusion criteria for this review during initial screening. The remaining (*n* = 23) studies were read in full by two authors (SN and AA). A total of 18 studies were excluded after reading full-text and their reason/s for exclusion were tabulated (Additonal file 3). Finally, five studies were found to meet the criteria for this systematic review and were thereafter included in data synthesis. A meta-analysis was not conducted due to the small number of included studies and heterogenous interventions and outcomes [40]. A PRISMA flow diagram was constructed, detailing the identification, screening and eligibility process (Fig. 1).

### Study characteristics

Five studies met the inclusion criteria and were included in this review. These included one step-wedged randomised trial with longitudinal sub-study [41, 42], one pragmatic randomised trial [43], one randomised controlled trial [44], and two pre-post longitudinal quasi-experimental studies [45, 46]. The included studies were published between 2012 and 2018 and were written in English language. The studies took place in a range of locations with widely varied demographic characteristics including: communities in the very remote regions of the Northern Territory [41, 42]; urban and regional areas of Western Australia [46]; as well as metropolitan and rural areas of South Australia [43, 44] and south east Queensland [45]. Three studies reported consultation and collaboration with the communities/populations of interest during the development of projects, and the employment of Indigenous staff in their implementation [42, 44, 45]. Loss to follow-up and poor attendance were a significant issue for several studies (*n* = 3) [41, 43, 45]. This was despite organisers offering increased flexibility and fiscal incentives aimed at increasing participation [41, 44].

The exact number of participants included in these studies was indeterminable due to the Brimblecombe et al. [42] study offering community-wide interventions. The authors of this study estimate the population of the communities in which the intervention was offered to be approximately 8515 persons, 95% of whom identify as Indigenous [42]. The total definitive number of Indigenous participants across all other studies was 902. This number included both men and women, though the majority of participants involved in all studies were female (*n* = 686 or 76.05%). Participants ranged in age from 18 to 74+ years. Two studies incentivised participation, with participants offered gifts, vouchers, or prizes for attendance at workshops/demonstrations or for the completion of questionnaires [41, 44].

### Intervention strategies and their effectiveness

There were two main types of intervention strategies among the studies: workshops/group education sessions [43–46] and reduction in the pricing of fruit and vegetables [42].

#### Workshops and group education

All of the studies included in this review provided some form of education to participants in order to improve their health literacy. The workshops or group education sessions included structured exercise classes [43, 45], nutrition/cooking workshops [43, 46], discussions/role-playing, presentations and other related learning activities [44].

Pettigrew et al. [46] recorded greater improvements in the self-reported confidence of Indigenous participants in their ability to buy healthy foods on a budget following attendance at workshop/s when compared to non-Indigenous participants (Mean = 0.74, SD = 1.17 among Indigenous participants and Mean = 0.53, SD = 1.05 among non-Indigenous participants). This occurred across all outcome measures with no modification of the intervention for cultural specificity.

The study conducted by Canuto et al. [43] also offered education on nutrition, though these workshops focussed on nutrition in relation to reducing the risk of cardio-metabolic disease specifically. The results of this education were assessed using anthropometric and biomedical measures. Attendance at a structured exercise and nutrition education program over a period of 12-weeks for Indigenous women with waist circumferences > 80 cm was found to contribute to a reduction in weight from baseline (Mean: 2.5 kg, 95% CI, 4.46–0.54) and BMI (Mean: 1.03 kg/m2, 95% CI, 1.79–0.27) [41]. Differences in other anthropometric measurements and biomedical markers were statistically insignificant, though this may be due to the relatively short follow up period of only 6-months.

On a similar theme, Mills and colleagues [45] also aimed to address risk of chronic cardiovascular disease in at-risk individuals through the offering of exercise groups and ‘yarning’ sessions (a form of circular dialogue used to build relationships and share information in Indigenous Australian cultures [48]). Like the Canuto et al. [43] study, outcomes were assessed by calculation of changes in biophysical measures, though this time over a very brief follow up period of just 12-weeks. Participation in the program described in this study was demonstrated to lead to a reduction in the weight of extremely obese participants (1.6 kg, 0.1–3.0 kg, 95% CI), improved distance able to be walked in six minutes (0.053 km, 0.01–0.07 km, 95% CI) as well as decreased blood pressure (BP) in participants who were hypertensive at baseline (11 mmHg, 3.2–18.8 mmHg, 95%CI) [45]. While the aim of these studies may have been to improve health literacy levels, it is possible that such results may have been influenced by other factors.

In the study conducted by Ju and colleagues [44], participation in workshops was demonstrated to improve overall oral health literacy (Mean change = 1.3, 1.1–1.6, 95% CI) [44]. The number of participants recognising the benefits of fluoridated drinking water saw the most significant elevation (94.0 intervention vs. 76.8 control, RR = 1.2, 95%CI). Other parameters saw improvements, though these were neither statistically significant nor repeated under all scenarios used in the study [44].

#### Reduction in the cost of fresh/frozen produce and low-sugar beverages

Brimblecombe et al. [42] demonstrated that the introduction of an in-store 20% discount on fruit, vegetable, water and artificially sweetened soft drinks was associated with a positive effect on sales of fruit and vegetables (12.7% increase in weight of fruit and vegetables purchased, 4.1–22.1, 95% CI). This effect was enhanced with the addition of consumer education at point-of-sale (increasing sales by a further 7.6%, 3.6–20.2, 95% CI) [42]. The perceived affordability of fresh produce was later also positively associated with healthy food choices and dietary changes in the population of interest [41].

### Outcomes

The results of all included studies demonstrate statistically significant improvement in at least one health literacy related outcome measure following participation in the intervention. However, each study assessed the effect of the intervention/s using different measures or tools with only one study using a measurement tool to assess health literacy [44].

One randomised controlled trial [44] utilised the specially formulated ‘Health Literacy in Dentistry’ scale to quantify changes in the primary outcome (health literacy related to oral health). This tool was specifically developed in collaboration with the population of interest for this study to ensure cultural safety and sensitivity [44]. Secondary outcomes of this intervention were also measured using scale to ascertain awareness of the social impacts of oral health, sense of personal control to maintain good oral health, oral health-related self-efficacy and general dental knowledge [44].

Both of the studies examining interventions to mitigate risk or impact of cardiovascular disease in at risk or affected individuals [43, 45], did so through implementation of structured group exercise classes, and measured outcomes through the collection biomedical and anthropometric data from participants.

Three studies offered some form of nutrition education as part of their broader interventions [42, 43, 46]. Outcome measures for each of these studies were however quite different; with Brimblecombe [42] measuring changes in purchases of fruits, vegetables and beverages; Canuto et al. [43] and Mills et al. [45] assessing outcomes using measurements of biophysical markers and anthropometry; and Pettigrew et al. [46] utilising self-reported improvements in knowledge and confidence to measure post-intervention outcomes. Using these varied measures, each study successfully generated quantifiable results from the intervention/s employed.

### Assessment of methodological quality

Three studies were assessed using the checklist for Randomised Control Trials [42–44] and two using the checklist for Pre-Post Quasi-Experimental studies [45, 46] produced by the JBI [47]. The results of the application of these quality assessment instruments have been reported descriptively here and can be found in tabulated version in Additional file 4 and 5. All eligible studies were included in this review irrespective of their methodological quality.

Each included study demonstrated partially adequate methodological quality. Whilst all five scored strongly when assessed using the JBI checklists, issues with participation in interventions and/or retention of participants in several [41, 43, 44]; as well as a lack of consultation with the population of interest and missed opportunities for co-design and collaboration in others [43, 46] detracted from otherwise generally robust methodological quality.

The randomised control trials [42–44] demonstrated adequate randomisation; blinding, treatment, outcome measurement; and suitable statistical analysis. One pre-post longitudinal study [46] also proved to be of partially adequate methodological quality, although it (and the study by Ju et al. [44]) relied solely on self-reported data for comparison of pre- and post-intervention, and therefore required participants to possess adequate literacy levels to complete questionnaires. The Pettigrew et al. [46] study also lacked post-intervention measurement of biophysical data which may have demonstrated actual behaviour change, though weakness was identified by the authors and may have been beyond the scope of the study.

The pre-post quasi-experimental study conducted by Mills and colleagues [45] was of strong methodological quality and measured biophysical markers at baseline and predetermined intervals throughout the study period to assess outcomes. This quality of this study was also bolstered by prioritised building of trust, promotion of community control throughout the designing and implementation of the intervention and through the involvement of Indigenous researchers.

The Canuto et al. [43] and Ju et al. [44] studies reported very poor attendance (with only around 40% attendance at workshops/classes for each), and high loss to follow-up. This substantially limited sample sizes, effectiveness of programs, and the amount of data collected. Ju et al. [44] utilised multiple imputation to compensate for missing data while the Canuto et al. [43] study reported only on available data. There was also some concern of possible sample contamination voiced by the authors of the oral health literacy trial, [44] as the pilot of the study had been held in close geographic proximity to the trial itself.

## Discussion

The purpose of this systematic review was to identify, appraise, and create a synthesis of interventions to improve health literacy amongst Indigenous Australian adults. Of the five studies that were included in this review, four included interventions to improve health literacy in relation to lifestyle factors and nutrition; and one addressed oral health literacy. Studies varied in size and scope from the involvement of thousands of individuals across multiple communities, to clusters of small groups with as few as one hundred participants involved in others. Interventions were commonly provided in the form of workshops or classes. Each included study was somewhat successful in fulfilling its objectives with statistically significant improvement in at least one outcome related to health literacy measured.

In order to increase the scope of this review and capture a broad range of interventions targeting all possible measures of health literacy, this review did not require the outcomes to be assessed using a specific health literacy measurement tool such as The Rapid Assessment of Health Literacy in Medicine (REALM), The Test of Functional Health Literacy in Adults (TOFHLA) or The Newest Vital Sign [49]. As none of these instruments have been found to comprehensively assess an individual’s true capacity [50] and have been criticised by some for over-simplifying health literacy [49], this review included all manner of outcome measures related to the improvement of knowledge, skills, attitudes, motivation or behaviour changes, self-efficacy or improved self-management, engagement with and use of available health care services as well as health outcomes; which may all be considered the result of enhanced health literacy. While changes in measurements of these outcomes may be impacted by other factors, they have been included for completeness as they are considered to be important aspects of health literacy able to be influenced by the application of intervention [5].

The interventions included in this review comprised oral health literacy workshops [44]; as well as the delivery of cooking, nutrition and exercise classes [43, 45, 46]. Attendance at education sessions such as these provides participants with the opportunity to gain knowledge; and may also enhance motivation through shared experiences and increased sense of social connection. Workshops have been successfully employed as a means to provide targeted interventions to improve health literacy among varied groups; including those with certain disease pathologies or risk factors, senior citizens, refugees, parents of paediatric patients, as well as among other Indigenous populations (New Zealand Māori, Native American, Taiwanese Aboriginals) [51–56].

The study by Brimblecombe and colleagues [42] was the largest included in this review, and measured the impact of a discount on fruit, vegetables, water and low-sugar beverages in twenty communities across very remote regions of Western Australia. This intervention increased health literacy in the target population by providing education, increasing awareness and facilitating an increase in the sale of healthier foods. The intervention led to an increase in the amount of fruits and vegetables purchased from stores during the period of the intervention and this affect appeared to persist following its completion. A modest added impact was noted with the addition of in-store education (aimed at improving health literacy related nutrition) which was randomly assigned to half of the communities involved in the study. These results are consistent with those of similar studies conducted amongst the general population in both New Zealand and the Netherlands [57, 58]. Other Australian research has also shown positive health benefits could possibly be derived from the imposition of revenue-neutral taxes on less healthy foods to subsidise fresh fruit and vegetables [59]. A similar strategy is already used in countries around the world, where a ‘sugar-tax’ on soft drinks has been implemented with the aim of combating obesity and related non-communicable diseases [60]. This strategy has demonstrated effectiveness by increasing public awareness about sugar consumption, decreasing purchases of sugar sweetened beverages (SSBs) and encouraging SSB manufacturers to decrease the sugar content of their products [60]. Pressure to implement such a tax in Australia is currently mounting [61], though any such measure must of course be applicable to the entire population and not target specific sub-groups.

Two studies included in this review [41, 44] utilised some form of incentive in order to encourage participation or reward/retain participants. Incentives are seen by many as a means to improve health outcomes [62], recognise the valuable contributions of individuals to the determination of robust research outcomes, and enhance recruitment and retainment in research [63]. Incentives offered to participants included vouchers, ‘prizes,’ fruit and vegetable baskets and intervention-related paraphernalia (such as water bottles, toothbrushes, dental mirrors etc.). The use of incentives in research and health promotion is commonly seen in successful studies involving Indigenous participants [64–66] and is soundly grounded in behaviour modification theory [67]. However, incentivising research remains a somewhat contentious strategy with some arguing that it may undermine autonomous decision-making by being coercive; compromise the integrity of results by inadvertent over-recruitment of those from lower-socioeconomic backgrounds; or that it may even reduce altruism and intrinsic motivation, with the possibility of becoming counter-productive and leading to decreased participation [63]. It is impossible to determine whether these factors were at play in the poor participation and significant loss to follow-up experienced in the studies included in this review.

Four of the five included studies reported some form of community consultation during development [42–45].. The studies utilising a participatory approach also employed Indigenous research officers, workshop facilitators and/or educators. Recently, other studies embracing continual collaboration and participant co-design have been successfully working to develop interventions to address health literacy among hospitalised patients [68] and to support new Aboriginal and Torres Strait Islander parents who have experienced complex trauma [69]. In the studies included here, this community involvement did not always translate into engagement and participation; with poor attendance at interventions and loss to follow-up reported even where investment in Indigenous consultation and involvement was considerable. This is particularly noted in the oral health literacy intervention conducted by Ju and colleagues [44].

The methodological quality of the included studies was partially adequate, with deficits found to have predominantly occurred due to missed opportunities for involvement of the population of interest during the research process, poor participation in interventions and significant loss to follow-up. The various randomised controlled trials [42–44] achieved strong results in terms of achievement of adequate randomisation, concealment of treatment groups and blinding. The pre-post studies [45, 46] also scored well on the standardised checklist employed [47]. Methods for measurement of outcomes across all studies were reliable, although Canuto et al. [43] reported some minor difficulties with the use of equipment to measure BP and waist circumference given the differing shapes of the participants involved in their study. Also, the use of self-reported questionnaires to measure outcomes [41, 44, 46] may have been problematic in that they assume a level of literacy and English proficiency sufficient to comprehend and appropriately respond to questions, which some participants may not have possessed. The Brimblecombe et al. [41] and Pettigrew et al. [46] studies also lacked any collection of biophysical data which may have been demonstrative of actual behaviour change post-intervention, though this was beyond the scope of these studies.

The results of this review highlight the profound complexities of addressing the health gap and the effects of existing interventions to improve health literacy among Indigenous Australians on addressing health inequities remain unknown. This can be seen to demonstrate the importance of ameliorating Indigenous self-determination through continual consultation and collaboration with community members from project inception to completion. In the wake of centuries of systemic devaluing of Indigenous Australian culture, the promotion of positive representatives and role models is critical to the development of a healthier, strength-based Indigenous identity [22].

It is important to consider also that all Aboriginals and Torres Strait Islander peoples themselves belong to smaller population groups, with over 500 distinct nations spread across the continent, each with its own world views, beliefs, traditions and many with distinct dialects/language [70]. None of the included studies report delivering interventions in the local dialects of participants, or report being responsive to the heterogeneity of Aboriginal and Torres Strait Islander peoples’ culture, though translators were offered to participants of the Pettigrew et al. study [46]. Differences in world view and language barriers between Aboriginal and Torres Strait Islander peoples and service providers are known to impede health literacy [71]. The use of First language could serve to empower communities, promote autonomy and create shared understandings between health care professionals and Indigenous communities that may otherwise be lost in translation [72]. Only one of the studies included in this review reports consideration of between or within-group diversity [45]. This may mean that the other interventions used could plausibly have been more or less effective when applied to different Indigenous nations [16], a factor that might have been mitigated by increasing collaboration and partnership with communities during the conception, design and implementation of research protocols.

### Strengths and limitations

This is the first systematic review to evaluate interventions to improve health literacy among Indigenous Australians. While the search encompassed six databases, including both peer-reviewed studies and grey literature and was supplemented by manual reference list searches, it is possible that some relevant studies may have been missed. This review was strengthened by the range of terms used to describe health literacy outcomes in the search of databases, leading to the capture of a broader variety of studies. However, this approach may have also limited the review by contributing to the heterogeneity of outcome measures seen among included studies, which hampered comparisons between interventions and made pooling of the results statistically to undertake a meta-analysis impossible. The use of these broad search terms may also have compromised the results of this review in that outcomes could have plausibly been influenced by other factors and not have been the result of increased health literacy. As with all systematic reviews, the results and conclusions depend on the quality of the published literature, with small sample sizes making it impossible to determine whether null findings represented a true lack of effect or simply reflected limitations in statistical power. The use of scales to measure health literacy has been criticised as being overly simplified and may limit the scope of studies if used in isolation [3, 46]. Whilst this review includes studies using health literacy scales, it also considers outcome measures related to the improvement of knowledge, skills, attitudes, motivation or behaviour changes, self-efficacy or improved self-management, engagement with and use of available health care services as well as health status outcomes to be indicative of a change in health literacy.

### Implications

To the best of our knowledge, this is the first systematic review that aims to synthesise the evidence on interventions to improve health literacy in Aboriginal and Torres Strait Islander populations. However, many opportunities remain for important future research. Future health literacy studies may benefit from an investigation of reasons for poor retention rates of Aboriginal and Torres Strait Island participants in research, and from an exploration of ways to modify approaches which may lead to improved participation throughout the research process. These may include encouraging the use of co-design and participatory approaches, use of language and culturally appropriate communication, the incorporation of community-specific perceptions of health and wellbeing in interventions and/or increasing engagement with Indigenous staff. Currently, researchers in public health use “adequate” and “inadequate” or “low” levels of health literacy which may have different meanings across different settings. The public health field will benefit if researchers clearly specify relevant cut-points of distinguishing different levels of health literacy. There may also be value in the creation of standardised instruments for the measurement of health literacy outcomes among Indigenous populations. It is also important for public health researchers to test skills-based health literacy measures as a focus of future research. It is ethically imperative for studies involving Indigenous Australians to openly, consistently and comprehensively collaborate with community members in all steps of the research process in order to strengthen Indigenous identity, increase self-determination and thereby enhance outcomes.

## Conclusion

This review found that limited evidence exists regarding interventions to improve health literacy in Aboriginal and Torres Strait Islander Peoples. Interventions were predominantly in the form of workshops, group education or reduction of the price of healthful foods and beverages. Whilst all interventions reported improvement in at least one health literacy outcome measure, the methodological quality was weakened by small sample sizes, poor attendance, and significant loss to follow-up. It is suggested that future research should involve substantial Indigenous community engagement in all aspects of the design and implementation of interventions, including careful consideration of culture, Indigenous concepts of health and well-being, as well as language. The reciprocal sharing of ideas, promotion of respect and enhancement of participation to strengthen autonomy and build empowerment among Aboriginal and Torres Strait Islander Peoples is key to improving health literacy and in doing so, reducing disparities and a building a healthier future.

## Supplementary Information

**Additional file 1.** PRISMA checklist.

**Additional file 2.** Search Strategy (Embase OVID).

**Additional file 3.** Reasons for exclusion of studies.

**Additional file 4.** JBI Critical Appraisal Checklist for Randomised Control Trials.

**Additional file 5.** JBI Checklist for Quasi-Experimental Studies.

## Data Availability

The datasets generated during and/or analysed during the current study are available from the corresponding author on reasonable request.

## Abbreviations

ABS: Australian Bureau of Statistics
AIHW: Australian Institute of Health and Welfare
ALPA: Arnhem Land Progress Aboriginal Corporation
ATSI: Aboriginal and Torres Strait Islander
COAG: Council of Australian Governments
BMI: Body Mass Index
BP: Blood Pressure
CRP: C-Reactive Protein
HbA1C: Glycated Haemoglobin
HeLD: Health Literacy in Dentistry
JBI: Joanna Briggs Institute
MeSH: Medical Subject Headings
MI: Multiple Imputations
NHMRC: National Health and Medical Research Council
OBS: Out Back Stores
PICOS: Population, Intervention, Comparator, Outcome, Study Design
REALM: Rapid Assessment of Health Literacy in Medicine
SSB: Sugar Sweetened Beverage
TOFHLA: Test of Functional Health Literacy in Adults
TSI: Torres Strait Islander
WHO: World Health Organization
