# Supplementary material for: Interventions to improve health literacy among Aboriginal and Torres Strait Islander Peoples: a systematic review
Source: BMC Public Health. 2021 Jan 30;21:248. doi: 10.1186/s12889-021-10278-x (PMC7847024; doi:10.1186/s12889-021-10278-x)
Supplement: Supplementary file 2 — Additional file 2. Search Strategy (Embase OVID). [file 12889_2021_10278_MOESM2_ESM.docx]

## Appendix 2. Search Strategy (Embase OVID)

| **MeSH Terms** | **Output** |
| --- | --- |
| 1. Health literacy.mp or health literacy/ | 13173 |
| 1. Indigenous people/ or Indigenous Australian/ | 7011 |
| 1. Australian Aborigine/ | 658 |
| 1. Torres Strait Islander/ | 726 |
| 1. Health education.mp. or exp. Health education/ | 331586 |
| 1. Consumer health information/ or patient information/ | 29837 |
| 1. Patient education mp. or patient education/ | 117877 |
| 1. Health knowledge mp. or attitude to health/ | 112736 |
| 1. Self efficacy mp. or self concept/ | 99908 |
| 1. 1 or 5 or 6 or 7 or 8 or 9 | 539998 |
| 1. 2 or 3 or 4 | 7518 |
| 1. 10 and 11 | 630 |
