# Supplementary material for: Interventions to improve health literacy among Aboriginal and Torres Strait Islander Peoples: a systematic review
Source: BMC Public Health. 2021 Jan 30;21:248. doi: 10.1186/s12889-021-10278-x (PMC7847024; doi:10.1186/s12889-021-10278-x)
Supplement: Supplementary file 3 — Additional file 3. Reasons for exclusion of studies. [file 12889_2021_10278_MOESM3_ESM.docx]

## Appendix 3. Reasons for exclusion of studies

| **Author/s and Year Published** | **Study Theme/s** | **Study Design** | **Reason/s for Exclusion** |
| --- | --- | --- | --- |
| Biggs et al. (2016) | Sexual health | PP longitudinal | Participants < 18 years |
| Brown et al. (2019) | Nutrition – healthy choices | Mixed Methods | Qualitative results |
| Doyle et al. (2016) | Evaluation of existing health promotion programs | Prospective data collection | Non-intervention |
| Eades et al. (2012) | Smoking cessation in pregnancy | RCT | Participants < 18 years |
| Gould et al. (2019) | Smoking cessation in pregnancy | Stepped Wedge RCT Pilot | Participants < 18 years  Qualitative results |
| Hu et al. (2018) | Chronic disease risk management | PP Quasi Experimental | Participants < 18 years |
| Hunter et al. (2007) | Evaluation of technological disadvantage | Data collection only | Non-intervention  Qualitative results |
| Ivers et al. (2006) | Smoking cessation | PP | Non-Indigenous participants |
| Lee et al. (2019) | Oral cancer | RCT | Non-Australian Indigenous |
| MacDonald et al. (2016) | Health promotion – nutrition | PP | Qualitative results |
| Marley et al. (2014) | Smoking cessation | RCT | Participants < 18 years |
| Nilson (2016) | Health promotion – women | Participatory action research | Qualitative results |
| Parmenter et al. (2019) | Chronic disease management | Focus groups (sub study) | Qualitative results |
| Phillips et al. (2014) | Health promotion | RCT | Participants < 18 years |
| Rowley et al. (2000) | Lifestyle modification | PP longitudinal | Participants < 18 years |
| Slade et al. (2011) | Dental health | Prospective Cluster RCT | Participants < 18 years |
| Treloar et al. (2018) | Sexual health | Data analysis only (sub study) | Qualitative |
| Valery et al. (2010) | Health education – asthma | RCT | Participants < 18 years |

PP: Pre-Post

RCT: Randomised Control Trial
