# Supplementary material for: Interventions to improve health literacy among Aboriginal and Torres Strait Islander Peoples: a systematic review
Source: BMC Public Health. 2021 Jan 30;21:248. doi: 10.1186/s12889-021-10278-x (PMC7847024; doi:10.1186/s12889-021-10278-x)
Supplement: Supplementary file 4 — Additional file 4. JBI Critical Appraisal Checklist for Randomised Control Trials. [file 12889_2021_10278_MOESM4_ESM.docx]

## Appendix 4. JBI Critical Appraisal Checklist for Randomised Control Trials

| Checklist Question | Brimblecombe et al. [43,44] | Canuto et al. [45] | Ju et al. [46] |
| --- | --- | --- | --- |
| 1. Was true randomization used for assignment of participants to treatment groups? | Y | Y | Y |
| 2. Was allocation to treatment groups concealed? | Y | Y | U |
| 3. Were treatment groups similar at the baseline? | Y | Y | Y |
| 4. Were participants blind to treatment assignment? | Y | Y | Y |
| 5. Were those delivering treatment blind to treatment assignment? | Y | U | Y |
| 6. Were outcomes assessors blind to treatment assignment? | Y | U | U |
| 7. Were treatment groups treated identically other than the intervention of interest? | Y | Y | Y |
| 8. Was follow up complete and if not, were differences between groups in terms of their follow up adequately described and analysed? | Y | Y (HLTF, PP) | Y (HLTF, PP, MI) |
| 9. Were participants analysed in the groups to which they were randomized? | Y | Y | Y |
| 10. Were outcomes measured in the same way for treatment groups? | Y | Y | Y |
| 11. Were outcomes measured in a reliable way? | Y | Y | Y (self-reported) |
| 12. Was appropriate statistical analysis used? | Y | Y | Y |
| 13. Was the trial design appropriate, and any deviations from the standard RCT design (individual randomization, parallel groups) accounted for in the conduct and analysis of the trial? | Y | Y | Y |

RCT: Randomised Control Trial Y: Yes

PP: Poor participation N: No

HLTF: High Loss to Follow-up U: Unclear

MI: Multiple Imputation used
