# Supplementary material for: Interventions to improve health literacy among Aboriginal and Torres Strait Islander Peoples: a systematic review
Source: BMC Public Health. 2021 Jan 30;21:248. doi: 10.1186/s12889-021-10278-x (PMC7847024; doi:10.1186/s12889-021-10278-x)
Supplement: Supplementary file 5 — Additional file 5. JBI Checklist for Quasi-Experimental Studies. [file 12889_2021_10278_MOESM5_ESM.docx]

## Appendix 5. JBI Checklist for Quasi-Experimental Studies

| Checklist Question | Mills et al. [48] | Pettigrew et al. [47] |
| --- | --- | --- |
| 1. Is it clear in the study what is the cause and what is the effect (i.e. there is no confusion about which variable comes first)? | Y | Y |
| 2. Were the participants included in any comparisons similar? | Y | Y |
| 3. Were the participants included in any comparisons receiving similar treatment/care, other than the exposure or intervention of interest? | Y | Y |
| 4. Was there a control group? | Y (‘pre’ data) | Y (‘pre’ data) |
| 5. Were there multiple measurements of the outcome both pre and post the intervention/exposure? | Y | Y |
| 6. Was follow up complete and if not, were differences between groups in terms of their follow up adequately described and analysed? | Y | Y |
| 7. Were the outcomes of participants included in any comparisons measured in the same way? | Y | Y |
| 8. Were outcomes measured in a reliable way? | Y | Y (self-reported) |
| 9. Was appropriate statistical analysis used? | Y | Y |

Y: Yes

N: No

U: Unclear
